# Activating enhancer-binding protein-2α induces cyclooxygenase-2 expression and promotes nasopharyngeal carcinoma growth

**DOI:** 10.18632/oncotarget.3215

**Published:** 2014-12-31

**Authors:** Dingbo Shi, Xiangsheng Xiao, Yun Tian, Lijun Qin, Fangyun Xie, Rui Sun, Jingshu Wang, Wenbin Li, Tianze Liu, Yao Xiao, Wendan Yu, Wei Guo, Yuqing Xiong, Huijuan Qiu, Tiebang Kang, Wenlin Huang, Chong Zhao, Wuguo Deng

**Affiliations:** ^1^ Sun Yat-sen University Cancer Center, State Key Laboratory of Oncology in South China, Collaborative Innovation Center of Cancer Medicine, Guangzhou, China; ^2^ Institute of Cancer Stem Cell, Dalian Medical University, Dalian, China; ^3^ Department of Pediatrics, Sun Yat-sen Memorial Hospital, Sun Yat-sen University, Guangzhou, China; ^4^ State Key Laboratory of Targeted Drug for Tumors of Guangdong Province, Guangzhou Double Bioproduct Inc., Guangzhou, China

**Keywords:** AP-2α, COX-2, p300, nasopharyngeal carcinoma

## Abstract

Activating enhancer-binding protein-2α (AP-2α) regulates the expression of many cancer-related genes. Here, we demonstrated a novel mechanism by which AP-2α up-regulated cyclooxygenase-2 (COX-2) expression to promote the growth of nasopharyngeal carcinomas (NPCs). High expression of AP-2α in NPC cell lines and tumor tissues from NPC patients was detected and significantly correlated with COX-2 expression. Overexpression of AP-2α and COX-2 in tumor tissues was associated with advanced tumor stage, clinical progression, and short survival of patients with NPCs. Knockdown of AP-2α by siRNA markedly inhibited COX-2 expression and PGE2 production in NPC cells. Exogenous expression of AP-2α up-regulated the COX-2 and PGE2. Knockdown of AP-2α also significantly suppressed cell proliferation in NPC cells in vitro and tumor growth in a NPC xenograft mouse model. Moreover, we found that p300 played an important role in the AP-2α/COX-2 pathway. AP-2α could co-localize and interact with p300 in NPC cells. Overexpression of the p300, but not its histone acetyltransferase (HAT) domain deletion mutant, promoted the acetylation of AP-2α and its binding on the COX-2 promoter, thereby up-regulated COX-2 expression. Our results indicate that AP-2α activates COX-2 expression to promote NPC growth and suggest that the AP-2α/COX-2 signaling is a potential therapeutic target for NPC treatment.

## INTRODUCTION

Cyclooxygenase catalyzes the formation of prostaglandin, which is the common precursor for synthesis of diverse prostaglandins and thromboxane [1]. There are two isoforms of cyclooxygenases: cyclooxygenase-1 (COX-1) and cyclooxygenase-2 (COX-2) [2]. COX-1 is expressed constitutively in most tissues and responsible for various physiological functions [3]. COX-2 is an immediate and early response gene that is rapidly induced by phorbol esters, growth factors, cytokines, and oncogenes [4]. COX-2 is a key enzyme in the systhesis of prostaglandin E2 (PGE2). Overexpression of COX-2 and PGE2 is recognized as a marker for tumor progression documented for colon [5], lung [6], glioma [7], pancreas [8], hepatocellular carcinoma [9] and breast cancer [10]. A role of COX-2 in tumor development and progression has been demonstrated by both overexpression and disruption of the COX-2 gene as well as application of the drugs blocking COX-2 expression [11]. This role of has primarily been attributed to the production of PGE2 in the tumor microenvironment [12]. The tumor derived PGE2 has been shown to act as a para-crine as well as an auto-crine factor to promote breast cancer progression and metastasis by multiple mechanisms, such as inactivation of host anti-tumor immune cells and stimulation of tumor cell migration, invasiveness and tumor-associated angiogenesis [13-16]. However, the excise mechanism of the regulation of COX-2 and its clinical significance in nasopharyngeal carcinomas (NPCs) remains unknown at present.

The diverse actions of COX-2 that result in tissue damage and tumor growth depend on COX-2 transcriptional activation. COX-2 promoter activation by pro-inflammatory mediators and mitogenic factors has been characterized considerably [17,18]. Some enhancer elements have been demonstrated to be essential for promoter activation of COX-2 by pro-inflammatory mediators [19,20]. Each pro-inflammatory mediator requires binding of a combination of different transactivators to their respective enhancer elements. For example, phorbol 12-myristate 13-acetate (PMA) increases binding of activator protein-1 (AP-1) to CRE and C/EBP to C/EBP elements while tumor necrosis factor (TNF-α) induces NF-κB binding to NF-κB sites [21,22].

Transcription co-activator p300 is involved in transcriptional regulation of COX-2 [23-25]. It is closely related to CREB (cyclic AMP–response element binding protein)–binding protein (CBP) with a high degree of sequence homology [26]. P300 and CBP share binding domains and histone acetyltransferase (HAT) activities [27]. They interact with DNA-bound transactivators and bind general transcription factors such as transcription factor IIB (TFIIB), thereby integrating the transcriptional signals from external stimuli. It is generally believed that they have similar functions and play important roles in gene expression.

AP-2α is a member of the AP-2 transcription factor family proteins, which includes five different isoforms known as: AP-2α, AP-2β, AP-2γ, AP-2δ and AP-2ε [28]. AP-2 factors orchestrate a variety of cell processes including apoptosis, cell growth, and tissue differentiation during embryogenesis [29]. AP-2 homo and hetero-dimers can activate transcription via GC-rich DNA sequences [30]. AP-2 has been shown to bind the palindromic consensus sequence 5′-GCCN3GGC-3′, found in various cellular and viral enhancers [31]. AP-2 family is known to exhibit both activating and repressing effects on target genes. A number of genes involved in cell growth, cell shape, cell movement, cell fate and cell communication are regulated by AP-2 family. Reduced or overexpressed AP-2α is often detected in several types of cancers, such as melanoma, prostate, breast, ovary, gastric, colon and bladder cancers [32-39], indicating that loss of AP-2α function-or AP-2α overexpression may contribute to tumorigenesis and development of tumor malignancy. However, the regulation of COX-2 by AP-2α in NPC cells and the biological role, prognostic value and clinical significance of AP-2α/COX-2 signalling, as well as its molecular mechanisms of actions in NPC growth and progression are unclear. In addition, whether the cooperation of p300 with AP-2α is essential for the transcription regulation of COX-2 in NPC cells remains unknown.

In the present study, we analyzed the expression of AP-2α and COX-2 in various NPC cell lines and tumor tissues and evaluated the relationship between AP-2α and COX-2 with the clinicopathological factors. We also investigated the regulation of AP-2α on COX-2 expression and tumor growth in human NPC cells *in vitro* and in a NPC xenograft mouse model, and identified the underlying molecular mechanisms. Our findings provide new insights into understanding the role of the AP-2α/COX-2 signaling pathway in NPC tumorigenesis and exploring the potential therapeutic targets for NPC treatments.

## RESULTS

### Overexpression of AP-2α and COX-2 in NPC cell lines

**Figure 1:**
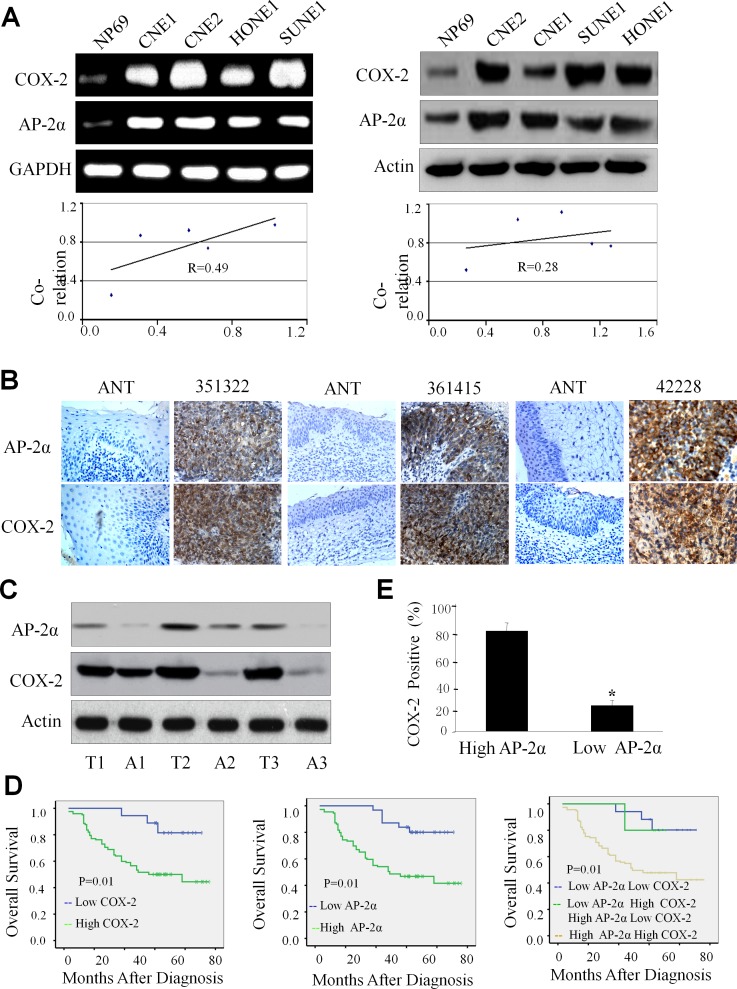
High expression of AP-2α and COX-2 in NPC cells and tumor tissues (A) The expression of AP-2α and COX-2 at mRNA *(left panel)* and protein levels *(right panel)* in various NPC cell lines was analyzed by RT-PCR and Western blot analysis, respectively. The correlations for the relative densities between AP-2α and COX-2 expression were analyzed *(lower panel)*. (B, C) The expression of AP-2α and COX-2 proteins in human NPC tumor tissues (T) and the adjacent non-cancer tissue (ANT) was detected by immunohistochemical (B) and Western blot analysis (C), respectively. (D) Kaplan–Meier analysis of survival of patients with NPCs expressing COX-2, AP-2α, or both proteins (*p*<0.001, log-rank test). (E) The association between AP-2α expression and COX-2 protein levels in tumor tissues from NPC patients.

We first detected the expression levels of AP-2α and COX-2 by RT-PCR and Western blotting analysis in nasopharyngeal carcinoma cells (CNE2, CNE1, HONE1 and SUNE-1) and normal nasopharyngeal epithelial cells (NP69). All four NPC cell lines had higher expression of AP-2α and COX-2 mRNA by comparison with the normal nasopharyngeal epithelial cell line NP69 (Fig. 1A, left panel). Western blot analysis also showed that the proteins of AP-2α and COX-2 were highly expressed in all NPC cell lines but not NP69 cells (Fig. 1A, right panel). The relative density was calculated by expression ratio of AP-2α or COX-2 to the internal control GAPDH or β-actin, and the results showed that the expression of AP-2α and COX-2 at mRNA and protein levels were positively correlated (Fig. 1A, lower panel).

### Overexpression of AP-2α and COX-2 in tumor tissues of NPC patients

Expression of AP-2α and COX-2 proteins were determined by immunohistochemical staining and Western blotting analysis in NPC tumor tissues and their adjacent non-cancer tissues. Immunohistochemical analysis showed that specific AP-2α staining was mostly found in the nuclei of the carcinoma cells. No specific AP-2α staining was observed in normal epithelial cells and in the surrounding stroma cells (Fig. 1B). COX-2 staining was mostly detected in the cytoplasm and nuclei of the tumor cells, and only a few scattered in filtrating lymphocytes and normal epithelial cells (Fig. 1B). By comparison with the adjacent non-cancer tissues, high expression of both AP-2α and COX-2 proteins were observed in tumor tissues from all three cases by Western blot (Fig. 1C, left panel).

### Positive correlation of AP-2α/COX-2 expression with clinicopathologic features in NPC patients

To gain further insight into the prognostic value of the AP-2α/COX-2 signaling pathway in NPC patients, the levels of AP-2α and COX-2 proteins were tested and compared between the tumor tissue samples and the adjacent non-tumor tissue samples. High positive AP-2α expression was localized to the nuclei in 143 resected tumor tissue samples (71.5%), whereas the remaining 57 cases displayed low levels of nuclei localization (28.5%). High positive COX-2 expression was localized to the cytoplasm and nuclei in 145 resected tumor tissue samples (72.5%), whereas the remaining 55 cases displayed low levels nuclei and cytoplasm localization (27.5%) (Table 1). Immunohistochemical determination of AP-2α levels was also statistically analyzed to identify its association with the clinicopathologic features of NPCs. As shown in Table 1, AP-2α expression was significantly correlated with clinical stage (P<0.001), T classification (P<0.001), N stage (P<0.001), distant metastasis (P<0.001), recurrence (P=0.034) and COX-2 expression (P< 0.001). However, there was no significant correlation between AP-2α expression and the age and gender of patients (P=0.576 and P=0.642). Additionally, COX-2 expression was also significantly correlated with clinical stage (P<0.001), T classification (P<0.001), N stage (P<0.001), distant metastasis (P<0.001) and recurrence (P=0.018). There was no significant correlation between COX-2 expression and the age and gender of patients (P=0.169 and P=0.745).

**Table 1:** Correlation between the expression of AP-2α with COX-2 and the clinicopathologic features in nasopharyngeal carcinomas

	All cases	AP-2α			COX-2		
		Low	High	P valuea	Low	High	P valuea
Sex				**0.642**			**0.745**
Male	145	40(27.6%)	105(72.4%)		44(30.3%)	101(69.7%)	
Female	55	17(30.9%)	38(69.1%)		18(32.7%)	37(67.3%)	
Age (years)				**0.576**			**0.169**
≤45	92	28(30.4%)	64(69.6%)		33(35.9%)	59(64.1%)	
>45	108	29(26.9%)	79(73.1%)		29(26.9%)	79(73.1%)	
Clinical stage				**0.001**			**0.001**
1	19	18(94.7%)	1(5.3%)		18(94.7)	1(5.3%)	
2	59	23(38.9%)	36(61.1%)		24(40.7%)	35(59.3%)	
3	79	10(12.7%)	69(87.3%)		11(13.9%)	68(86.1%)	
4	43	6(13.9%)	37(86.1%)		9(20.9%)	34(79.1%)	
T classification				**0.001**			**0.001**
1	14	11(78.6%)	3(21.4%)		11(78.6%)	3(21.4%)	
2	62	24(38.7%)	38(61.3%)		24(38.7%)	38(61.3%)	
3	86	14(16.3%)	72(83.1%)		19(22.1%)	67(77.9%)	
4	38	8(21.1%)	30(79.9%)		8(21.1%)	30(78.9%)	
N classification				**0.001**			**0.001**
0	37	20(54.1%)	17(45.9%)		20(54.1%)	17(45.9%)	
1	79	24(30.4%)	55(69.6)		25(31.6%)	5468.4%)	
2	56	8(14.3%)	48(85.7%)		8(14.3%)	48(85.7%)	
3	28	5(17.9%)	23(82.1%)		9(32.1%)	19(67.9%)	
Distant metastasis				**0.001**			**0.001**
0	153	55(35.9%)	98(64.1%)		57(37.3%)	96(62.7%))	
1	47	2(4.3%)	45(95.7%)		5(10.6%)	42(89.4%)	
Recurence				**0.034**			**0.018**
0	18	9(50%)	9(50%)		10(55.6%)	8(45.4%)	
1	182	48(26.4%)	134(74.1%)		52(28.6%)	130(71.4%)	

### Association of AP-2α/COX-2 expression with poor prognosis of NPC patients

To investigate the biological and clinicopathologic significance of AP-2α and COX-2 expression in NPC patients, we analyzed the relationship between NPC patient survival time and the AP-2α/COX-2 protein expression in tumor tissues from the NPC patients by immunohistochemical staining. The median survival time of the 200 NPC patients was 57.5 months (range 9–76 months). The overall survival in the low COX-2 expression group were significantly improved compared to the high expression group (P=0.01, Fig. 1D, left panel). Additionally, the high AP-2α expression group had significantly low overall survival than the group with low AP-2α expression (P=0.03, Fig. 1D, middle panel). Moreover, low expression of both AP-2α and COX-2 had high overall survival rate than the double high expression of AP-2α and COX-2 (P=0.01, Fig. 1D, right panel).

We further evaluated the potential correlation between the expression of AP-2α and COX-2 in NPC cohort. The results showed a positive correlation between the expression of AP-2α and COX-2 in NPCs. For the 143 NPC cases, 137 case showed high expression of AP-2α, an average of 96.6% of the carcinoma cells stained positive with COX-2 protein; the percentage was significantly higher than that (85.9%) in the remaining 57 cases NPCs with 49 cases of high expression of AP-2α (P<0.001, Fig. 1E).

### Upregulation of COX-2 and PGE2 by AP-2α in NPC cells

**Figure 2:**
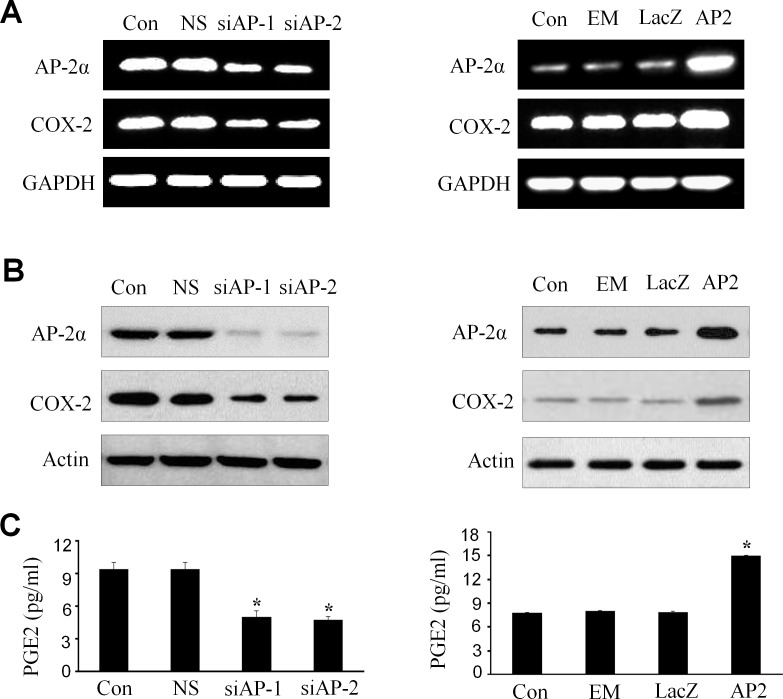
Upregulation of COX-2 and PGE2 by AP-2α in NPC cells The CNE2 cells were transfected with AP-2α siRNA (siAP1 and siAP2, 100 nM) (*left panel*) or AP-2α expressing vectors (AP2, 4 ug) (*right panel*). After 72 hours, the expression of AP-2α and COX-2 at mRNA (A) and protein levels (B) in NPC cells was detected by RT-PCR (A) and Western blot (B), respectively, and the release of PGE2 in cell culture supernatants was measured by ELISA in the treated CNE2 culture supernatants (C). The levels of GAPDH and β-actin were used as sample loading controls respectively. The non-specific scramble siRNA (NS) and the plasmid of LacZ or empty vector (EM) were used as the control groups for transfection experiments. Con, the control group treated with cell culture medium. The data are presented as mean ± SD of three tests. *, P < 0.05,

Since AP-2α expression positively correlated with COX-2 levels in NPCs, we next determined the effect of AP-2α knockdown or overexpression on the expression of COX-2 at mRNA and protein levels in NPC cells by RT-PCR and Western blot analysis, and on the release of PGE2 in cell culture media by ELISA assay. Transfection with AP-2α siRNA (siAP-1 or siAP-2, 100 nM) dramatically inhibited the expression of COX-2 mRNA (Fig. 2A, left panel) and proteins (Fig. 2B, left panel). Similarly, treatment of NPC cells with AP-2α siRNA (siAP-1 or siAP-2, 100 nM) also significantly inhibited the release of PGE2 protein in cell culture media (Fig. 2C, left panel). By contrast, overexpression of AP-2α by transfection with the AP-2α expressing plasmid dramatically promoted the expression of COX-2 mRNA (Fig. 2A, right panel ) and proteins (Fig. 2B, right panel) as well as the release of PGE2 in cell culture media (Fig. 2C, right panel).

### Regulation of cell proliferation by AP-2α/COX-2 signaling in NPC cells

**Figure 3:**
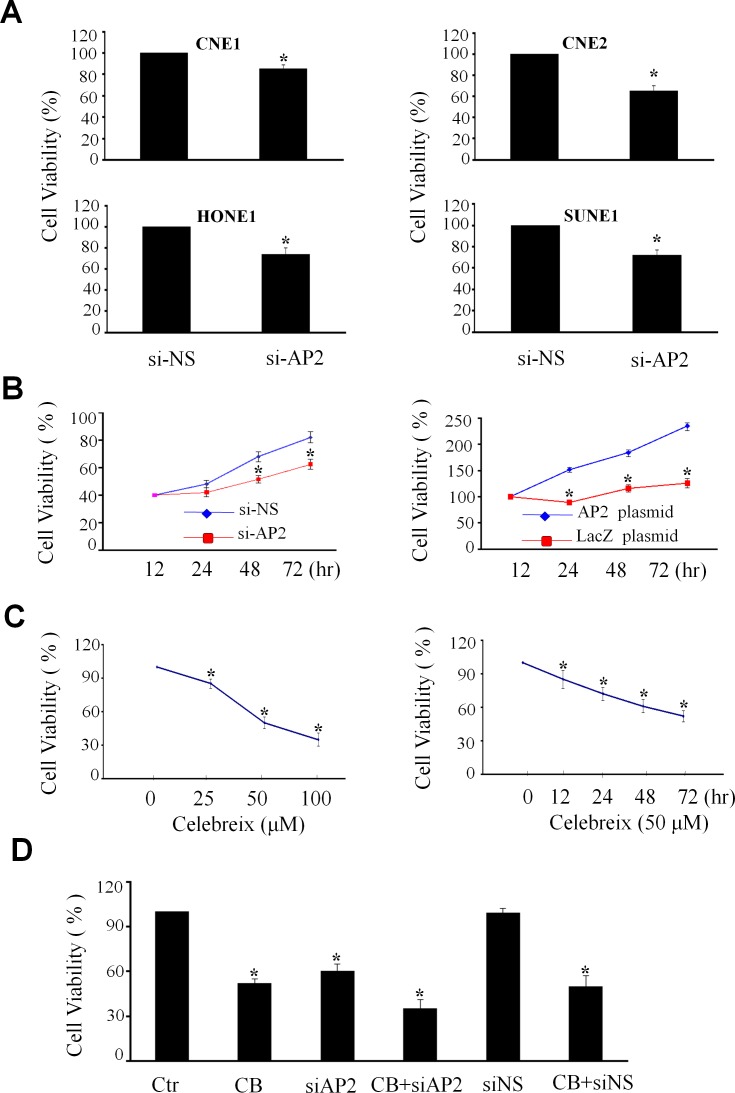
Regulation of NPC cell proliferation by AP-2α (A) Four NPC cell lines (CNE1, CNE2, HONE1 and SUNE1) were transfected with AP-2α siRNA (100 nM). After 72 hours, the proliferation activity of cells was detected by MTT assay. (B) The CNE2 cells were transfected with AP-2α siRNA (si-AP2, 100 nM) *(left panel)* or AP-2α expressing vector (4 ug) *(right panel)*. At 12, 24, 48 and 72 hours, the cell viability was detected by MTT assay. The non-specfic scramble siRNA (siNC) and the LacZ plasmid were used the controls. (C) CNE2 cells were treated with Celebrex (CB) at various concentrations (25, 50, 100 uM) for 72 hours *(left panel)* or at 50 uM for different time *(right panel)*. The cell viability was detected by MTT. (D). The CNE2 cells were transfected with AP-2α siRNA (siAP2) or the non-specfic scramble siRNA (siNC) at the doses of 100 nM for 24 hours, and then treated with Celebrex (CB) (50 uM). After 48 hours, the cell viability was analysis by MTT. All the data are presented as mean ± SD of three tests. *, p<0.05.

To determine the role of AP-2α/COX-2 signaling pathway in the regulation of the growth of NPC cells, we examined the effect of knockdown of AP-2α by Dotap-based AP-2α siRNA nanoparticles (si-AP2) on cell viability in various kinds of human NPC cell lines, including high differentiated cell line CNE1, low differentiated cell line CNE2 and the other two cell lines SUNE1 and HONE1, by MTT analysis. The results showed that knockdown of AP-2α expression by AP-2α siRNA (si-AP2, 100 nM) for 72 hours significantly inhibited cell viability when compared with the transfection with the non-specific siRNA (si-NS, 100 nM) and mock control groups, resulting in a 20% to 30% inhibitions in cell viability in the four kinds of NPC cell lines (Fig. 3A). Among these cell lines, CNE2 was dominantly inhibited by AP-2α siRNA in a time-dependent manner (Fig. 3B, left panel). Furthermore, we transfected CNE2 cell with an AP-2α expressing plasmid, and showed that overexpression of AP-2α significantly promoted NPC cell proliferation by comparison with the transfection with the LacZ control vector (Fig. 3B, right panel).

To further confirm AP-2α controls NPC cell growth via the modulation of the COX-2 signaling, we next detected the effect of Celebrex (CB), a COX-2-selective inhibitor, on NPC cell growth. Treatment with Celebrex considerably reduced the growth of CNE2 cells in a dose-dependent (Fig. 3C, left panel) and a time-dependent manner (Fig. 3C, right panel). Transfection of AP-2α siRNA did not significantly enhanced the Celebrex-mediated inhibition of cell growth (Fig. 3D). These results indicate that the AP-2α-mediated regulation of NPC cell growth may be partially through the regulation of COX-2 signaling.

### Regulation NPC tumor growth by AP-2α/COX-2 signaling in a xenograft mouse model

**Figure 4:**
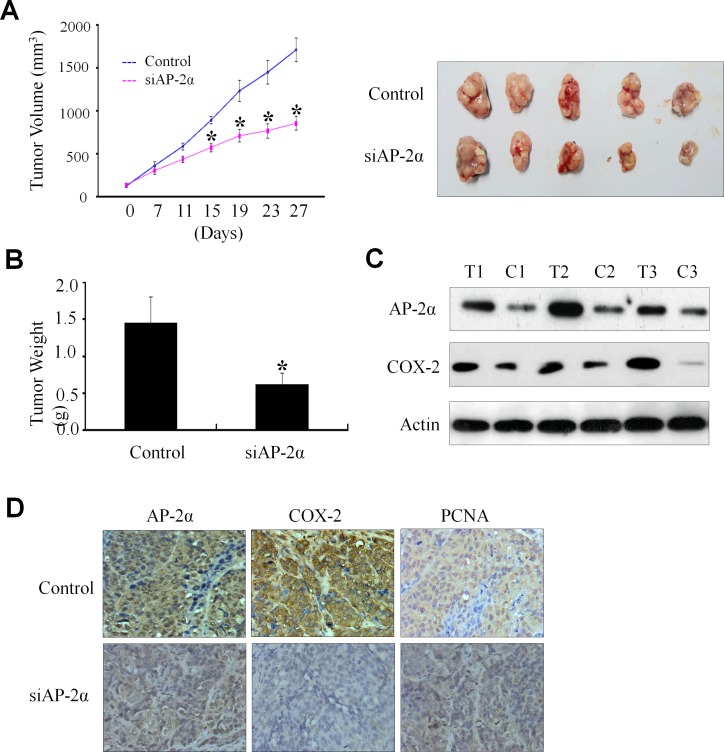
Inhibition of tumor growth by AP-2α siRNA in a xenograft mouse model The Dotap-nanoparticle-encapsulated AP-2α siRNA (si-AP2) and non-specific scramble siRNA (si-NS) were injected into the tumor regions of mice. Day 0 corresponds to 2 weeks after inoculation of CNE2 cells, and the first treatment was performed when tumor volume reached 150-160 mm^3^. Tumor diameters were measured at a regular interval of 4 days for up to 27 days with a digital caliper, and the tumor volume was calculated (A, *left panel*). The xenografts were harvested at 27 days after treatment. The pictures of the tumors were taken (A, *right panel*), and the weights of the tumors were analyzed (B). The expression levels of AP-2α, COX-2 and PCNA proteins in tumor tissues were detected by Western blot (C) and immunohistochemical staining (D). T, the tumors from the si-AP2-treated group; C, the tumors from the si-AP2-treated group. The data are presented as mean ± SD of three tests. *, *p*<0.05, significant differences between AP-2α siRNA (si-AP2) groups and the non-specific scramble siRNA (si-NS) groups. n=7 mice/group. Magnification, ×400.

The significant association of the AP-2α/COX-2 signaling with NPC cell survival and clinical outcome led us to further verify the essential role of AP-2α in regulating NPC growth and the expression of COX-2 in NPC mouse model *in vivo.* The CNE2 cells were injected subcutaneously into nude mice. After 2 weeks, visible tumors had developed at injection sites (mean tumor volume=150 mm^3^). The Dotap-nanoparticles encapsulating AP-2α siRNA (si-AP2) were then injected 6 times at a regular interval of 4 days for up to 27 days. Treatment with AP-2α siRNA (si-AP2) significantly inhibited the tumor volume as compared with the non-specific control siRNA treatment (si-NS) (Fig. 4A, left panel). The xenografts were harvested and the weights of the tumors were analyzed at 27 days after treatment. As shown in Fig. 4A (right panel) and Fig. 4B, AP-2α siRNA (si-AP2) treatment significantly inhibited tumor growth and the weights of tumors.

We also analyzed the levels of AP-2α proteins in tumors by immunochemical staining and showed a significant inhibition of AP-2α expression by si-AP2 (Fig. 4C). To verify the regulation of AP-2α on COX-2 expression in NPC tumor growth *in vivo*, we carried out a systematic analysis to test the effect of si-AP2 on COX-2 expression in tumors by Western blot (Fig. 4C) and immunohistochemical analysis (Fig. 4D). Consistent with the *in vitro* data, AP-2α knockdown (Fig. 4C, T1-T2-T3 and Fig. 4D) significantly inhibited COX-2 expression by comparison with those treated with the control scrambled siRNA (Fig. 4C, C1-C2-C3 and Fig. 4D). We also examined the effect of AP-2α knockdown on the expression of PCNA, an important indicator for tumor growth. Silencing of AP-2α expression in the NPC nude mice significantly reduced PCNA expression levels of the tumors as compared with the control groups (Fig. 4D). These *in vivo* results were consistent with those observed *in vitro* and confirmed the regulatory role of AP-2α in NPC tumor growth by partially controlling COX-2 expression.

### Binding of AP-2α to COX-2 promoter in NPC cells

**Figure 5:**
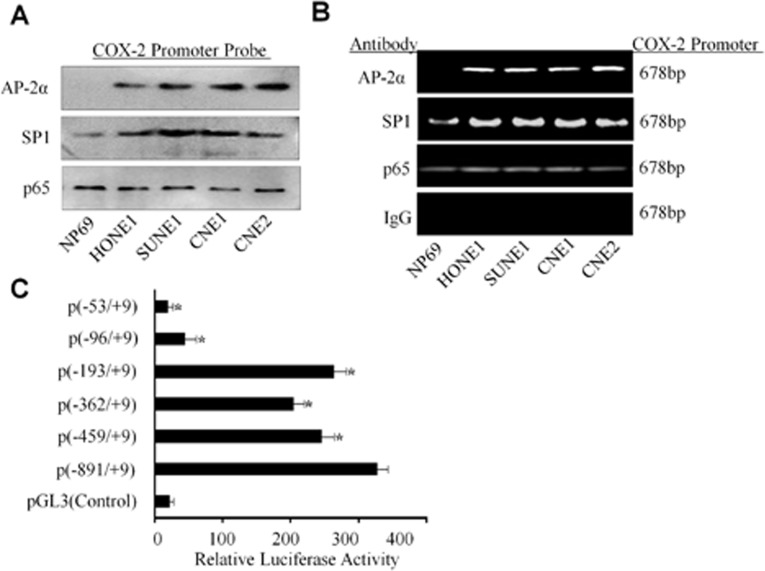
Binding of AP-2α to the COX-2 promoter in NPC cells (A) The binding of the transactivators SP1, p65 NF-κB and AP-2α to the biotin-labeled COX-2 promoter probe (−891/+9) was analyzed by pulldown and Western blot analysis. (B) Binding of the transactivators SP1, p65 NF-κb and AP-2α to the COX-2 promoter in chromatin structure was analyzed by ChIP assay. IgG was used as a negative control. (C) The CNE2 cells were co-transfected with AP-2α expressing vector and the multiple COX-2 promoter constructs. Transfection of pGL3 was used a control. The data are presented as mean ± SD of three tests. *, P < 0.05.

We next analyzed the underlying mechanism of AP-2α in the regulation of COX-2 transcription. We analyzed and identified a set of putative transcription factor binding site in the *COX-2* proximal promoter, including multiple NF-κB, SP1, and a single AP-2 binding site. To further demonstrate the COX-2 promoter-binding proteins of the human COX-2 promoter in NPC cells, a DNA fragment which is −891 to +9 nucleotides relative to the transcriptional start site of COX-2 was labeled by biotin in its 3′ and 5′ terminal. The normal nasopharyngeal epithelium cell NP69 and multiple NPC cell lines nucleus proteins were respectively extracted and mixed with the COX-2 promoter DNA probe and the streptavidin-agarose beads. The pulled down protein complexes were detected respectively by Western blot using the antibodies against SP1, p65 NF-κB and AP-2α proteins. The results showed detectable SP1 and NF-κB in all the cells, but the AP-2α protein only appeared in NPC cell lines but not in the normal epithelium cell line NP69 (Fig. 5A). The binding of AP-2α and the other trans-activators was further evaluated by ChIP assay (Fig. 5B). The results revealed an increased SP1, p65 NF-κB and AP-2α binding to the chromatin COX-2 promoter region in the NPC cancer cells than that in normal cells. A non-immune rabbit IgG was used as a negative control, and COX-2 promoter region was undetectable (Fig. 5B).

To define AP-2α binding to the regions of the COX-2 promoter, transient transfections were performed with human COX-2 promoter constructs and AP-2α expressing plasmids. As shown in Fig. 5C, AP-2α treatment caused an increase in COX-2 full promoter (−891/+9) activity, whereas the promoter activity was reduced in the absence of SP1 site (−459/+9) or NF-κB site (−362/+9 and −193/+9). However, deletion of the AP-2 site (−96/+9) caused a marked inhibition of the COX-2 promoter activity. Deletion of the SP1, NF-κB and AP-2 binding sites (−53/+9) almost completely blocked the COX-2 promoter activation (Fig. 5C).

### Co-localization and interaction of p300 with AP-2α in NPC cells

**Figure 6:**
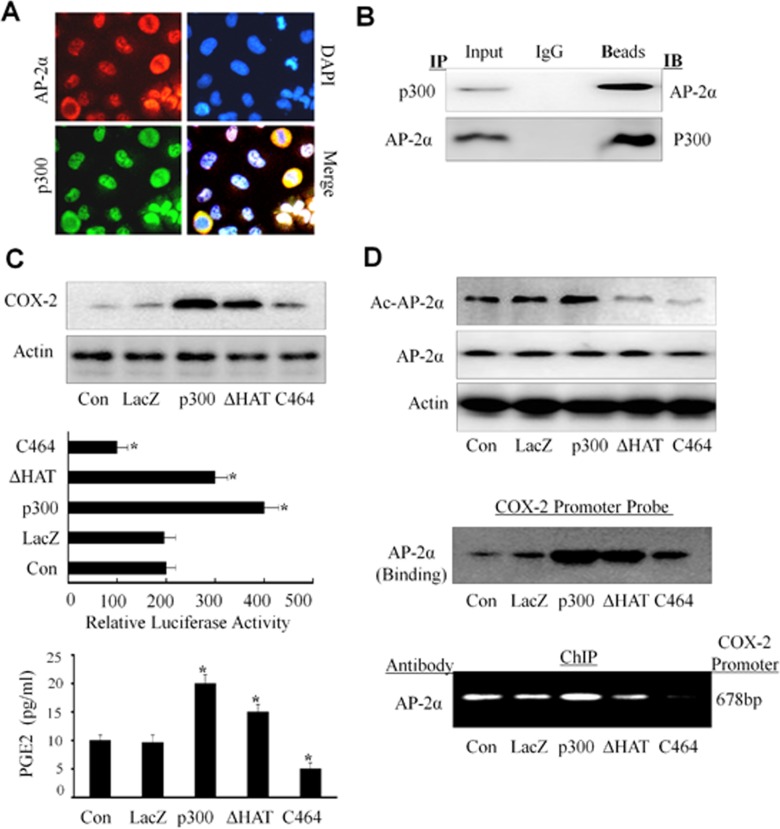
Up-regulation of COX-2 expression by p300 in NPC cells (A) Co-localization of AP-2α and p300. The CNE2 cells were doable stained with fluorescein isothiocyanate and rhodamine and examined by a confocal microscopy. The data were the representative cells with positive nuclei staining for AP-2α and p300. DAPI was used to stain the cell nuclei. (B) Interaction of AP-2α with p300. Co-Immunoprecipitation (IP) was performed using an anti-AP-2α or anti-p300 antibody, respectively, and the interaction of AP-2α with p300 in CNE2 cells was analyzed by Western blot. (C, D) The CNE2 cells were transfected with the expressing plasmids containing p300 or its HAT deletion mutant (ΔHAT) or COX-2 promoter-driven luciferase, or treated with the p300 inhibitor C646 (10 uM). After 48 hours, and the expression of COX-2 protein (C, *upper panel)* or the COX-2 promoter activity (C, *middle panel*) were analyzed. The secretion of PGE2 in CNE2 cell culture supernatants was also tested by ELISA (C, *lower panel*). The levels of the acetylated or total AP-2α proteins were detected by Western blot (D, *upper panel*). The binding of AP-2α to the biotin-labeled COX-2 promoter probe (D, *middle panel*) or the COX-2 promoter in chromatin structure (D, *lower panel*) was detected by Western blotting and ChIP analysis, respectively. The data are presented as mean ± SD of three tests. *, P < 0.05.

It has shown that p300, a transcriptional co-activator, controls COX-2 expression and transcriptional activation [41]. A key role of p300 in gene transactivation is conferred by its binding to a myriad of trans-activators that bound to their specific regulatory elements on the promoter. We next determined whether there was an association between AP-2α and p300 in NPC cells. We first analyzed the co-localization of p300 with AP-2α in CNE2 cells by immunofluorescence analysis. As shown in Fig. 6A (left panel), both p300 (green) and AP-2α (red) were detected in cell nucleus and cytoplasm, but most staining were observed in cell nucleus. The co-localization analysis showed that AP-2α and p300 had the same sub-cellular localization in NPC cells.

We next determined whether AP-2α and p300 can physically interact by immunoprecipitation assay. The nucleus proteins from CNE2 cells were extracted and incubated with anti-p300 or anti-AP-2α antibody, respectively, and then immunoprecipitated with protein A/G-agarose beads. The immune complexes were analyzed by Western blot. The results showed that p300 protein was seen in the immune complexes precipitated by the antibodies against AP-2α, and also AP-2α protein was detected in the immune complexes precipitated by the antibodies against p300 (Fig. 6B). These results suggest a physical interaction of AP-2α with p300 in NPC cells.

### Acetylation of AP-2α by p300 in NPC cells

The protein availability of p300 in nuclear extracts is considered to be a limiting step for transcription factor in gene regulation. We next detected the effect of p300 on AP-2-mediated COX-2 expression in NPC cells. The results showed that over-expression of p300 by transient transfection of CNE2 up-regulated COX-2 protein expression (Fig. 6C, upper panel) and the PGE2 release (Fig. 6C, lower panel). Also, over-expression of p300 by transfection with its plasmid and the COX-2 full promoter (−891/+9) construct increased COX-2 promoter activity (Fig. 6C, middle panel). However, inhibition of p300 by its specific inhibitor C646 significantly reduced COX-2 protein expression, PGE2 release and COX-2 promoter activity (Fig. 6C).

To ascertain that the regulation of COX-2 by AP-2α needs the help of p300, we evaluated the effect of p300 overexpression or inhibition on the expression of AP-2α and its acetylation in CNE2 cells. P300 overexpression by transient transfection resulted in a increase in acetylation of AP-2α proteins (Fig. 6D, upper panel), but did not affect the expression of AP-2α (Fig. 6D, upper panel). Furthermore, we found that transient transfection of the CNE2 cell with p300 plasmids effectively increased the binding AP-2α to the COX-2 promoter when compared with the non-transfected cells or the control vector (LacZ) group (Fig. 6D, middle panel) by pulldown analysis.. Furthermore, inhibition of p300 activity by its inhibitor C464 in CNE2 cells reduced the binding of AP-2α to the COX-2 promoter (Fig. 6D, middle panel) and the acetylation of AP-2α protein (Fig. 6D, upper panel). The same results were also detected in the ChIP assay (Fig. 6D, lower panel). Thus, our results indicate that p300 plays a more dominant role in mediating and regulating AP-2α transcription activity in COX-2 expression.

### Downregulation of AP-2α/COX-2 signaling by p300 HAT in NPC cells

The p300 HAT is known to acetylate chromatin histone thereby increasing accessibility to trans-activators [44]. We were therefore interested in determining whether overexpression of p300 HAT would influence the acetylation of AP-2α, the binding of AP-2α to COX-2 promoter, and the promoter activity of COX-2. Transfection with the p300 HAT deletion mutant effectively reduced the acetylation of AP-2α (Fig. 6D, upper panel), the binding AP-2α to COX-2 promoter (Fig. 6D, middle and lower panels), and the promoter activity of COX-2 (Fig. 6C, middle panel). Furthermore, transfection of the p300 HAT deletion mutant plasmid also decreased COX-2 protein expression (Fig. 6C, upper panel) and PGE2 release (Fig. 6C, lower panel) compared with the transfection with the wild-type p300 plasmid (p300). Our results therefore show that p300 HAT plays a key role in the AP-2α/COX-2 signaling pathway in NPC cells.

## DISCUSSION

In this study, we demonstrated that AP-2α and COX-2 were highly expressed in both human NPC cancer tissues and the NPC cell lines, and found that high expression of AP-2α and COX-2 in NPC patients was correlated with clinicopathologic features, such as clinical classification, T stage, N stage, metastasis and recurrence. We also showed that the patients with high expression of AP-2α and COX-2 have a short survival. These results reveal that the AP-2α/COX-2 signaling plays an important role in nasopharyngeal carcinoma progression and development. Our results were consistent with the previous studies. COX-2 expression was elevated in human colorectal adenocarcinoma and other tumors, including breast cancer, cervical cancer, prostate cancer, and lung cancer, etc. Genetic knock-out or pharmacological inhibition of COX-2 has been shown to protect against experimentally-induced carcinogenesis [45]. Transgenic mice overexpressing COX-2 in mammary glands, skin or stomach develop malignancies of these organs [46]. Knock-out of COX-2 gene suppresses the development of intestinal tumors or skin papillomas [47]. Taken together, these results indicate that up-regulation of the AP-2α/COX-2 play a key role in NPC tumorigenesis, progression and development.

In the experiments to shed light on the mechanisms responsible for the up-regulated COX-2 expression in NPC cells and tumor tissues, the DNA-protein binding assay was performed to identify the potential binding proteins in NPC cell lines by a biotin-labeled COX-2 promoter DNA probe. We found the nuclear transcription factors SP-1, p65 NF-κB and AP-2α was pulled down, but the AP-2α protein was only pulled down in NPC cells but not in the nasopharyngeal epithelial cells. ChIP analysis was also performed to prove the binding of these factors to the cis-regulatory element located in COX-2 promoter. The results showed that only AP-2α specifically bound to COX-2 promoter in the NPC cells.

The human COX-2 promoter contains multiple transcription factor binding sites. The previous reports showed NF-κB p65 and p50 could bind to the NF-κB sites located in the COX-2 promoter region in human epithelial cells and mouse inflammatory cell when stimulated with IL-1, PMA and LPS [40-41]. The reports also demonstrated that AP-1 nuclear protein can bind to the CRE site located in the COX-2 promoter region in human mammary cells [48]. While CRE binding activity was attenuated in the presence of 100-fold excess of unlabeled AP-1 oligonucleotides in cervical cancer cells [49]. PPARγ ligand treatment reduces the binding activities of both AP-1 and NF-κB nuclear proteins in cervical cancer cells [50]. On the other hand, PPARγ ligands had little effect on AP-2 binding in the COX-2 promoter, indicating the different binding sites within COX-2 promoter region for different transcriptional factors. To determine whether these effects were responsible for AP-2α-mediated control of COX-2, transient transfection experiments were performed with a series of COX-2 promoter reporter constructs in which the binding sites for NF-κB and/or AP-2 nuclear protein were deleted and the plasmids of AP-2α or the siRNA of AP-2α in NPC cell. The results demonstrated that down-regulation of COX-2 by knocking down AP-2α and up-regulation by overexpressed AP-2α was predominantly mediated by the trans-activating activity of AP-2α on the COX-2 promoter AP-2 site. This finding reveals that AP-2α binding to AP-2 site plays a major role in mediating up-regulation of COX-2 expression in NPC cells. In contrast, the modulation of COX-2 expression by PPARγ in macrophages was largely mediated by trans-activating factor NF-κB binding to the COX-2 promoter NF-κB site [32]. Our study showed that the regulation of COX-2 through AP-2α is cell-type specific and may also be dependent on developmental processes.

The present data and previous findings have demonstrated that alteration of COX-2 levels can be mediated by a diverse group of seemingly unrelated natural, dietary, and synthetic compounds that have been shown to bind to and activate PPARγ. Our studies demonstrated that AP-2α can control expression of COX-2 in NPC cells. P300 is a predominant isoform in human fibroblasts and murine macrophages. Its binding to COX-2 promoter is up-regulated by pro-inflammatory mediators [40]. Our following study demonstrated the relationship between p300 and AP-2α in controlling COX-2 expression in NPC cells. To our knowledge, this is the first report of a selective involvement of p300 and AP-2α in COX-2 transcriptional regulation in human NPC cells.

Consistent with our previous reports, COX-2 transcription was found to be regulated by p300 in NPC cells. Overexpression of p300 augmented COX-2 promoter activity, COX-2 protein level and PGE2 release in NPC cells. We also found the co-localization of AP-2α with p300 and their interaction physically in NPC cells. Overexpression of p300 can increase the acetylated levels of AP-2α and enhance the binding of AP-2α to the AP-2 site located in COX-2 promoter but not affect the levels of AP-2α. We also performed molecular analysis by mutations of p300 in order to understand the requirement of various binding domains and HAT for COX-2 transcriptional activation, the acetylation of AP-2α, and AP-2α binding to the COX-2 promoter. Deletion of the core HAT domain of p300 results in a marked reduction in p300-mediated COX-2 transcriptional activation, COX-2 expression and the levels of acetylation AP-2α. These results are attributed to a major role that p300 HAT plays in opening up the chromatin structure at the COX-2 promoter region to make the enhancer elements in COX-2 promoter accessible to trans-activators. It is interesting to note that p300 HAT also plays a role in regulating the acetylation of AP-2α and enhancing its following recruitment to the COX-2 promoter. It has been reported that p300 acetylates several trans-activators such as p53, GATA-1, p50 and p65, thereby increasing their binding activity. These results are consistent with our research.

In conclusion, our study showed that there was a positive regulation between COX-2 and AP-2α gene expression in human NPC cancer cells. The ability of AP-2α to control COX-2 gene expression may be mediated predominantly through its acetylation by p300 and its binding to the COX-2 promoter. Knockdown or inhibition of AP-2α could suppress NPC cell proliferation *in vitro* and tumor growth *in vivo*. Since both COX-2 and AP-2α display significantly positive correlations with clinical-pathological factors of NPC, our results suggest that the AP-2α/COX-2 signaling pathway may be a useful target for the therapy and diagnosis of NPCs.

## MATERIALS AND METHODS

### Cell culture

The human NPC cell lines CNE1 (high differentiated), CNE2 (low differentiated), as well as HONE1, SUNE1 and the immortalized normal nasopharyngeal cell line (NP69) were cultured in DMEM medium, supplemented with 10% fetal bovine serum (FBS) (HyClone, Logan, UT), 100 U/ml penicillin and 100 μg/ml streptomycin (Sigma). Cells were maintained in a humidified atmosphere and 5% CO_2_ at 37°C. The cell lines have bot been authenticated by the author.

### NPC tumor Samples

NPC cancer samples were collected from patients with different histological types squamous cell carcinoma and adjacent non-carcinoma tissues. Histologically tissue samples were obtained from patients which were diagnosed with nasopharyngeal carcinoma undergoing electric nasopharyngoscopy at Sun Yat-Sen University Cancer Center (Guangzhou, China). Samples were stored at −80°C until analysis. Informed consent was obtained from each patient. This study was approved by the Committees on Human Rights in Research at Sun Yat-Sen University Cancer Center.

### Patient tissue specimens

This study was conducted on a total of 200 paraffin-embedded NPC samples, which were histologically and clinically diagnosed from the Cancer Center, Sun Yat-Sen University, between 2008 and 2012. For the use of these clinical materials for research purposes, prior patient's consent and approval from the the Committees on Human Rights in Research were obtained. The disease stages of all the patients were classified or reclassified according to the UICC system. Clinical information of the samples is described in detail in Table 1. Patients included 155 males and 45 females, of ages ranging from 18 to 82 years (mean, 45.9 years). The median follow-up time for overall survival was 57.5 months for patients still alive at the time of analysis, and ranged from 9 to 76 months. A total of 41 (20.5%) patients died and 35 (17.5%) patients experienced metastasis during follow up.

### Plasmids

The COX-2 promoter constructs ligated to luciferase (−891/+9, −459/+9, −362/+9, −193/+9,−96/+9 and−53/+9) has been reported previously [40-41]. The expressing vectors containing full-length p300 (p300) and its HAT deletion mutant (p300ΔHAT, Δ1472-1522) were used in the experiments.

### Transfections and luciferase assay

The transfection was performed using Lipofectamine 2000 reagent as previously described [42]. Renilla luciferase activity was measured using the DUAL-luciferase reporter assay kit. The expressed luciferase activity was measured in a luminometer. All of the vehicle controls were considered as 100%.

### RT-PCR

Total RNA was prepared from primary human nasopharyngeal carcinoma tissues and cultured cell lines by using Trizol Reagent (Life Technologies) according to the manufacturer's instructions. The sequences of PCR primers were: for AP-2α sense primer (5′-ATATCCGTTCACGCCGATCC-3′), antisense (5′-CGACCCGGAACTGAACAGAA-3′); for COX-2 sense (5′-ATAACCCCGCCAAAAGGGG-3′), antisense (5′-AGGAACAGCATGCAGGTAGC-3′); and for GAPDH sense (5′-CCATGGAGAAGGCTGGGG-3′), antisense (5′-CAAAGTTGTCATGGATGACC-3′). The RT-PCR was carried out as described (42).

### Western blot analysis

The incubated cells were disrupted in lysis buffer. The proteins were electroblotted onto a polyvinylidene difluoride (PVDF) membrane. The membrane was incubated with the antibodies against COX-2, AP-2α, SP-1, NF-κB and p300 and followed by the horseradish peroxidase (HRP)-conjugated anti-primary host IgG antibody (diluted 1:5,000). Immunoreactive bands were visualized with a Western blot detection system (Kodak IBM 4000 system, USA).

### DNA-protein binding assay

Binding of the protein to trans-activator of COX-2 promoter DNA complexes was assayed by a technique as described previously [40-41]. The biotinlabeled double-stranded oligonucleotide probes were synthesized by PCR based on human COX-2 promoter sequence −891 to +59. The binding assay was performed by mixing 400 μg uclear extracts, 4 μg biotin-labeled DNA oligonucleotides, and 40 μl streptavidin agarose beads. The mixture was incubated at room temperature for 2 hour with shaking. Beads were then pelleted down by centrifugation and washed with PBS. The binding proteins were separated on 12% PAGE followed by Western blot analysis probed with antibodies against AP-2α.

### MTT assay

Cell viability was determined by the MTT assay (Roche Diagnosis, Indianapolis, IN). Briefly, The NPC cell lines seeded in 96-well plates (2000 cells/well) were treated with si-RNA of AP-2α and (or) plasmids of AP-2α and P300 at the indicated doses. Cell viability was determined.

### Chromatin immunoprecipitation (ChIP)

The ChIP assay was done as previously described [43]. PCR amplification using specific COX-2 promoter primers: Forward primer,5′-CTGTTGAAAGCAACTTAGCT-3′ (−709 to −690); and Reverse primer, 5′-AGACTGAAAACCAAGCCCAT-3′ (− 32 to −51). The resulting product of 678 bp was separated by 1.2% agarose gel.

### Co-immunoprecipitation

Interaction of p300 with AP-2α transcription factor was determined by immunoprecipitation. Nuclear extract proteins (800 μg) prepared from CNE2 were incubated with a specific rabbit polyclonal antibody to AP-2α or p300 or a nonimmune rabbit IgG (Cell Signaling Technology), at a final concentration of 4g/mL each, overnight at 4°C. The immune complex was pulled down by protein A/G plus agarose (Santa Cruz Biotechnology), and after washing with RIPA buffer (50 mM Tris-HCL [pH 7.5], 150 mM NaCl, 1 mM EDTA, 1 mM PMSF, 1g/ml leupeptin, 5g/ml aprotinin, 1% Nonidet P40, 0.5%sodium deoxycholate, and 0.1% sodium dodecyl sulfate) 3 times, the immunoprecipitated proteins were separated by SDS–PAGE and analyzed by Western blotting using a p300 antibody or AP-2α antibody.

### PGE2 analysis by ELISA

The concentration of PGE2 in the supernatant was measured with an ELISA kit according to the manufacturer's instructions. PGE2 was measured using a VersaMax ELISA microplate reader.

### Determination of acetylated AP-2α

AP-2α in nuclear extracts was immunoprecipitated with a specific antibody of AP-2α (Cell Signaling Technology) and the immunoprecipitate was pulled down with protein A/G agarose beads. After extensive washing, COX-2 proteins were separated in a 12% SDS-PAGE system and the acetylated AP-2α was detected with a monoclonal antibody against acetyl-lysine.

### Immunofluorescence and confocal microscopy

The Cells were fixed with 4% paraformaldehyde (w/v) for 20 min, quenched for 20 min with 50 m M NH4 Cl in PBS and permeabilised with 0.2%. (w/v) saponin in PBS for 20 min. The saturation step was performed for 20 min in PBS containing 1% bovine serum albumin (BSA) and 0.2% saponin (w/v). Cells were then incubated overnight with the primary AP-2α and P300 antibodies diluted in PBS containing 1% BSA and 0.2% saponin. After PBS washings, cells were incubated for 1 h with secondary fluorescein isothiocyanate or tetra methyl rhodamine isothiocyanate (TRITC)-conjugated antibodies. AP-2α and p300 protein localization was assessed using a Leica confocal microscopy (Model TCS-NT). Files of microphotographs were processed with the Adobe Photoshop 5.0 software.

### Immunohistochemistry

Immunohistochemistry was done to study altered protein expression in human NPC tissues. In brief, paraffin-embedded specimens were cut into 4-μm sections and baked at 65°C for 30 min. The sections were de-paraffinized with xylenes and rehydrated. Sections were submerged into EDTA antigenic retrieval buffer and microwaved for antigenic retrieval. The sections were treated with 3% hydrogen peroxide in methanol to quench the endogenous peroxidase activity, followed by incubation with 3% bovine serum albumin to block the nonspecific binding. Rabbit polyclonal anti-AP-2α and COX-2 (1:200; CST) was incubated with the sections overnight at 4°C. For negative controls, the primary antibody was replaced by normal rabbit serum. After washing, the tissue sections were treated with biotinylated anti-rabbit secondary antibody (Cell Signaling Technology), followed by further incubation with streptavidin horseradish peroxidase complex (Cell Signaling Technology). The degree of immunostaining of formalin-fixed, paraffin-embedded sections was reviewed and scored by two independent observers. The proportion of the stained cells and the extent of the staining were used as criteria of evaluation. For each case, at least 1,000 tumor cells were analyzed and the percentage of positively nuclear stained tumor cells was recorded. For each sample, the proportion of AP-2α and COX-2-expressing cells varied from 0% to 100%, and the intensity of nuclear staining varied from weak to strong. One score was given according to the percent of positive cells as: < 5% of the cells:1 point; 6–35% of the cells:2 point; 36–70% of the cells:3 point; >71% of the cells: 4 point. Another score was given according to the intensity of staining as negative staining: 1 point; weak staining (light yellow): 2 point; moderate staining (yellowish brown): 3point; and strong staining (brown): 4 point. A final score was then calculated by multiple the above two scores. If the final score was equal or bigger than four, the tumor was considered high expression; otherwise, the tumor was considered low expression.

### Xenograft mouse model

To determine the effect of AP-2α siRNA on NPC tumor growth in a xenograft model, CNE2 cells (1 × 10^6^) were inoculated subcutaneously into the flank of the nude mice. Once palpable tumors were observed, tumor volume measurements were taken every four days using calipers. The tumor volume was calculated using the following formula: V = (length×width^2^)/2. Body weights were also recorded. two weeks after injection, the mice were randomized into 2 groups (5 mice/group). Group 1 received injection with *In Vivo* Ready nonspecific siRNA, and group 2 with *In Vivo* Ready AP-2α siRNA. Dotap-nanoparticles encapsulated siRNA duplexes were injected into the tumors using insulin syringes at a concentration of 10 μg of siRNA/50mm^3^ of tumor volume. All two groups were treated twice a week for 4 weeks. Upon termination, tumors were harvested and weighted. Animal experiments were approved by the Animal Research Committee of Sun Yat-Sen University Cancer Center and were performed in accordance with established guidelines.

### Evaluation of AP-2α silencing efficiency and COX-2 expression in xenograft tumor tissues

Tumor tissues from the above treated animals were collected and placed in 10% formalin and embedded in paraffin for below analysis. One sections were used for the extraction of proteins to assess the impact of AP-2α siRNA *in vivo* on COX-2 by Western blot. The rest were embedded for stained with COX-2 to investigate its expression. A negative control was obtained by replacing the primary antibody with a normal rabbit or mouse IgG. The immunoreactivity positive cells from each of the differently treated tumor tissue sections were measured at 200x magnification using a light microscope.

### Confocal microscopy

The cells were seeded onto coverslips in a 6-well plate, fixed with freshly prepared 3.5% paraformaldehyde for 30 min, and permeabilized with 0.1% Triton X-100 for 10 min. After 1 hr incubation with 3% BSA/0.1% Triton X-100/PBS, the cells were treated with primary anti-COX-2(1:200) or anti-p300 (1:200) antibody overnight at 4°C.. The cells were washed and then incubated with DyLight488-conjugated donkey anti-rabbit secondary antibody (1:500) for 30 min at RT. After several additional washing steps, the coverslips were mounted in VECTASHIELD mounting media with DAPI (Vector Labs, Burlingame, CA, USA). The results were visualized using XI81 confocal microscopy (Olympus, Japan).

### Statistical analysis

Statistical analysis was performed by using the SPSS16.0 statistical software package. Strong AP-2α and COX-2 immunoreactivity was assessed for the association with clinicopathologic variables such as gender, age and pathologic TNM stage by using the Pearson Chi-Square test. Survival curves were calculated from the date of surgery to the time of death related to NPC or to the last follow-up observation. Kaplan Meier curves were calculated for each relevant variable and for AP-2α and COX-2 expression. Differences in survival times among patient subgroups were analyzed by the log-rank test. Statistical significance was determined with Student's t test (two-tailed) comparison between two groups of data set. P < 0.05 in all cases was considered statistically significant
